# 3D-Printed and Biofabricated Scaffolds for Osteoporotic Bone Defect Repair: Mechanical Adaptation, Redox Control, Senescence Modulation and Osteoimmunomodulation

**DOI:** 10.3390/bioengineering13091088

**Published:** 2026-09-19

**Authors:** Sedeek Mosaid, Yousif Jihad, Nuala Pepper, Mohamed Elnemr, Ashok Marudanayagam, Paul Lee

**Affiliations:** Orthopaedic Surgery Department, United Lincolnshire Hospitals NHS Trust, Lincoln LN2 5QY, UKdr.mohamed.a.elnemr@gmail.com (M.E.);

**Keywords:** osteoporosis, bone defect, bone tissue engineering, three-dimensional printing, biofabrication, scaffold, oxidative stress, cellular senescence, osteoimmunomodulation, mechanobiology

## Abstract

Fragility fracture and osteoporotic bone loss represent an increasing global burden in ageing populations: the Global Burden of Disease Study 2021 estimated 172.79 million new fractures, 453.31 million prevalent cases, and 25.18 million years lived with disability in 2021, yet the resulting defects are still managed with grafts and scaffolds validated in healthy bone. Osteoporotic bone is not merely rarefied normal bone but a distinct regenerative microenvironment, in which we propose that oxidative stress, cellular senescence, and loss of osteogenesis-coupled type-H vasculature act as a mutually reinforcing triad, superimposed on an adipogenically biassed progenitor pool, blunted osteocyte mechanotransduction and immunosenescence. Additive manufacturing and biofabrication permit patient-specific geometry and spatial and temporal programming of mechanical and biological cues. This review appraises four interdependent design axes—mechanical adaptation through functionally graded, trabecular-mimetic architectures; redox regulation; senescence modulation; and osteoimmunomodulation coupled to vascularisation—distinguishing study type, disease specificity and author-proposed hypotheses. The most direct clinical signal is mechanical: in a retrospective cohort of 163 lumbar interbody fusion levels, printed porous titanium was associated with subsidence in 5.5% of levels versus 24.4% for solid titanium. By contrast, almost all redox, senescence and immunomodulatory scaffold evidence remains preclinical: osteoporosis-specific evidence relies heavily on ovariectomised rodents, and much of the rest comes from non-osteoporotic models. Within the searches described, we identified no completed clinical evaluation with reported results of a multifunctional scaffold in an osteoporosis-defined cohort. As the authors’ proposed conceptual framework rather than an established result, we suggest that these axes be engaged sequentially rather than additively, and that timing, dose and spatial distribution may matter more than the number of bioactivities.

## 1. Introduction

The Global Burden of Disease Study 2021 estimated 172.79 million new fractures worldwide in 2021, with 453.31 million prevalent cases and 25.18 million years lived with disability. Although age-standardised rates declined between 1990 and 2021, absolute case numbers and years lived with disability continued to rise. More new fractures occurred in men than in women (95.12 million versus 77.66 million), although age-specific fracture incidence in women exceeded that in men beyond the ages of 55–59 years [1]. Hip fractures illustrate this trajectory: across 4,115,046 cases in 19 countries and regions, the age- and sex-standardised incidence declined in most settings. However, the absolute annual number is projected to nearly double between 2018 and 2050, with a more substantial increase in men (2.4-fold) than in women (1.7-fold), and post-fracture anti-osteoporosis treatment rates range from 11.5% to 50.3% by country [2].

The same burden drives demand for reconstruction of osteoporotic bone loss [3]. Similar reconstructive needs arise in comminution, non-union, peri-implant osteolysis, revision arthroplasty, and tumour or infection surgery.

Repair is more difficult in osteoporotic bone, not only because there is less bone. Fracture healing is delayed in both oestrogen-deficiency and glucocorticoid-induced osteoporosis, which impair intramembranous and endochondral ossification differently [4]. Autografts, the reference standard, are limited by donor-site morbidity and finite supply [5], and their osteogenic yield depends on host biology, which is compromised by osteoporosis. Allografts avoid donor morbidity but incorporate unpredictably. Rigid metallic implants have an elastic modulus far exceeding that of bone, creating stress-shielding concerns in implant design [6] that may be particularly undesirable where bone stock is poor. Even highly osteoinductive adjuncts expose the limits of a purely additive approach: in a segmental defect model, supraphysiological bone morphogenetic protein-2 (BMP-2) induced heterotopic ossification and inflammation, highlighting the importance of dose and delivery vehicle; the relative contributions of dose, carrier, and local context remain unresolved [7].

Additive manufacturing and biofabrication offer qualitatively distinct capabilities, including patient-specific external geometry from computed tomography data, internal architectures graded to approximate trabecular bone, and spatial and temporal control of biological cues that passive grafts cannot provide. Some of these advances are already clinical. In transforaminal lumbar interbody fusion, a retrospective cohort of 124 patients across 163 interbody levels reported subsidence of any grade in 24.4% of solid-titanium levels versus 5.5% of printed-porous levels, with osteoporosis or osteopenia being an independent predictor of subsidence grade on multivariate analysis [8]. Printed constructs can also be tailored to osteoporosis: 3D-printed porous Ti6Al4V scaffolds co-delivering an osteogenic cue (BMP-2) and an anti-resorptive cue (osteoprotegerin, OPG) enhanced osseointegration under experimentally induced osteoporotic conditions [9]. However, many standard devices are not specifically designed for or prospectively validated in osteoporosis-defined populations and do not address redox, senescence, and immune biology, which are implicated in impaired repair [3,10].

**Figure 1:**
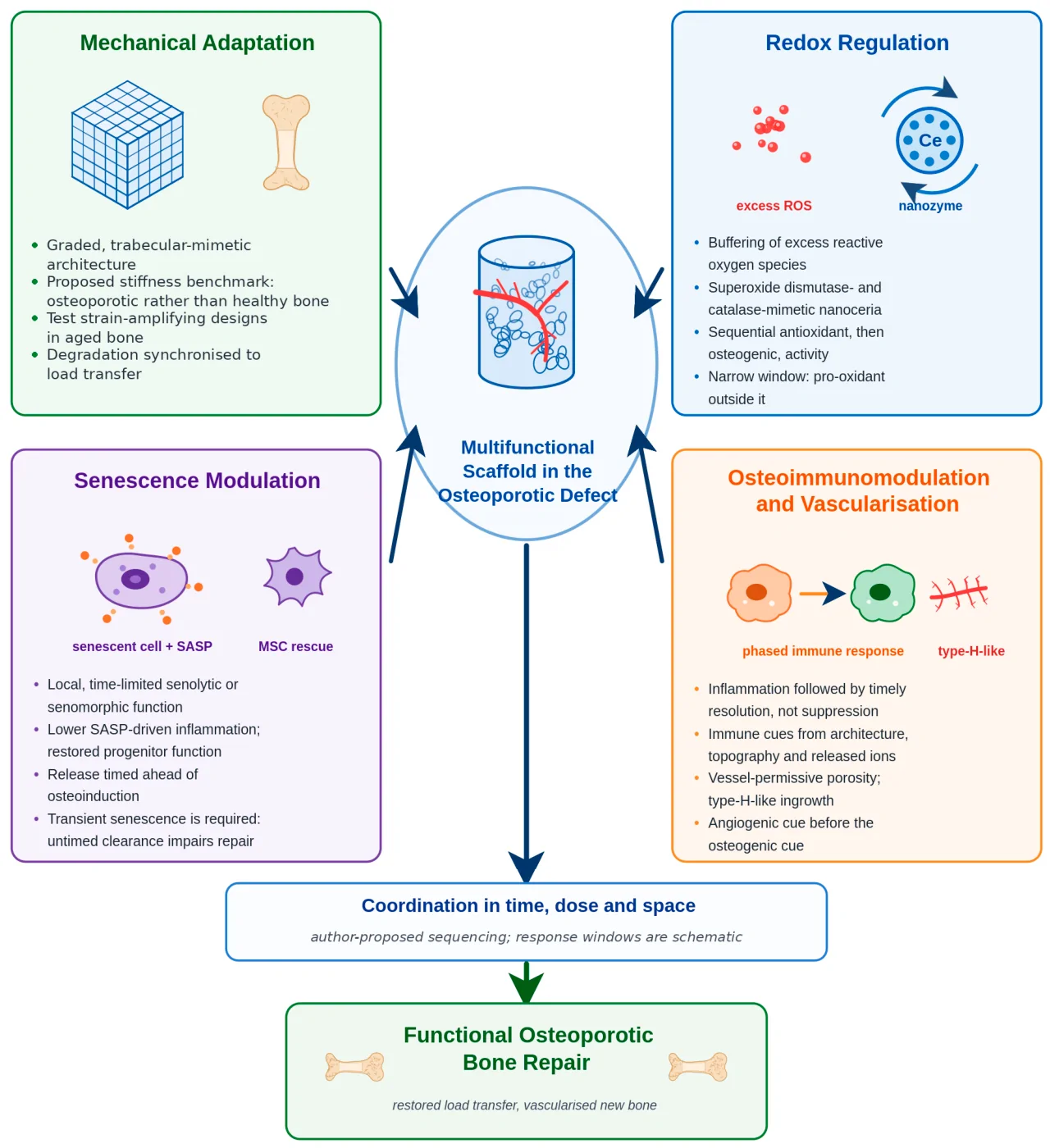
The four interdependent design axes for a scaffold intended for osteoporotic bone. The four axes converge on one construct implanted into an osteoporotic defect. The sequential framework and inverted-U window shown are author-proposed hypotheses, not experimentally established relationships.

Therefore, a scaffold optimised for healthy bone may be mismatched to osteoporotic bone. Recent reviews have addressed parts of this problem separately: reviews of 3D-printed biomaterials for osteoporosis have concentrated on materials and fabrication [3], reviews of immune regulation and osteoimmunomodulation in aged bone have concentrated on the inflammatory and immunosenescent niche [10]. The redox–senescence axis of skeletal ageing, meanwhile, has been reviewed as biology rather than as a set of scaffold-design requirements [11,12]. To our knowledge, none of these integrates mechanical adaptation, redox regulation, senescence modulation, and immunovascular coupling into a single design framework for osteoporotic bone, or distinguishes systematically between tested evidence and design hypothesis. The necessity for the present review follows from that gap: multifunctional constructs are already being fabricated, but without a disease-specific framework specifying which functions are needed, in what order, and on what evidence. This review makes three contributions. First, it draws oxidative stress, cellular senescence, and type-H vascular decline together in a design-relevant framework. While the redox–senescence axis and its role in skeletal ageing are well established [11,12], we add type-H vascular decline as a third component, proposing the resulting triad as a scaffold-design hypothesis rather than an established mechanism. Second, it organises the four axes into a time-dependent framework, addressing them sequentially rather than simultaneously. Third, it distinguishes evidence type and directness throughout and uses the inverted-U as a design heuristic rather than a universally demonstrated dose–response (Figure 1). The objectives of this review are therefore: (1) to characterise the osteoporotic defect as a distinct regenerative microenvironment; (2) to appraise additive manufacturing and biofabrication strategies against the four design axes, labelling the setting and directness of the supporting evidence; and (3) to propose a testable, time-sequenced design framework together with the studies required to evaluate it.

## 2. Review Methodology

This is a narrative rather than a systematic review; no protocol was registered, and no meta-analysis was conducted. PubMed/MEDLINE, Scopus, and Web of Science were searched until August 2026, combining terms for the disease context (osteoporosis, osteoporotic bone defect, ovariectomised), fabrication technology (three-dimensional printing, additive manufacturing, biofabrication, bioprinting, and scaffold), and the four biological axes examined here (mechanotransduction, oxidative stress, cellular senescence or senolytic, osteoimmunomodulation, type-H vessel or angiogenesis). ClinicalTrials.gov and the WHO International Clinical Trials Registry Platform were additionally searched for registered scaffold-based interventions relevant to osteoporosis; only completed studies with reported results were considered clinical evidence. Inclusion was limited to English-language journal articles and reviews; conference abstracts and non-English publications were therefore not considered, and earlier landmark studies were identified through citation tracking. Records were screened by title and abstract and retained if they addressed scaffold design, fabrication, biology, or clinical outcomes relevant to osteoporotic bone repair. Indirect evidence—from non-osteoporotic defect models, from systemic therapies of mechanistic relevance, and from the implant, dental, and fracture-healing literature—was eligible for inclusion but was recorded as indirect and is separated from direct osteoporosis-defect scaffold evidence throughout the text and tables. Evidence descriptors report study type and directness—in vitro, small-animal, large-animal, human observational, clinical trial, or established practice—rather than a single ordinal hierarchy; ‘mechanistically inferred’ and ‘author-proposed (untested)’ identify concepts not directly tested. The limitations of this approach, including the absence of a PRISMA-style screening log, are presented in Section 11.

## 3. Osteoporotic Bone as a Distinct Regenerative Microenvironment

**Figure 2:**
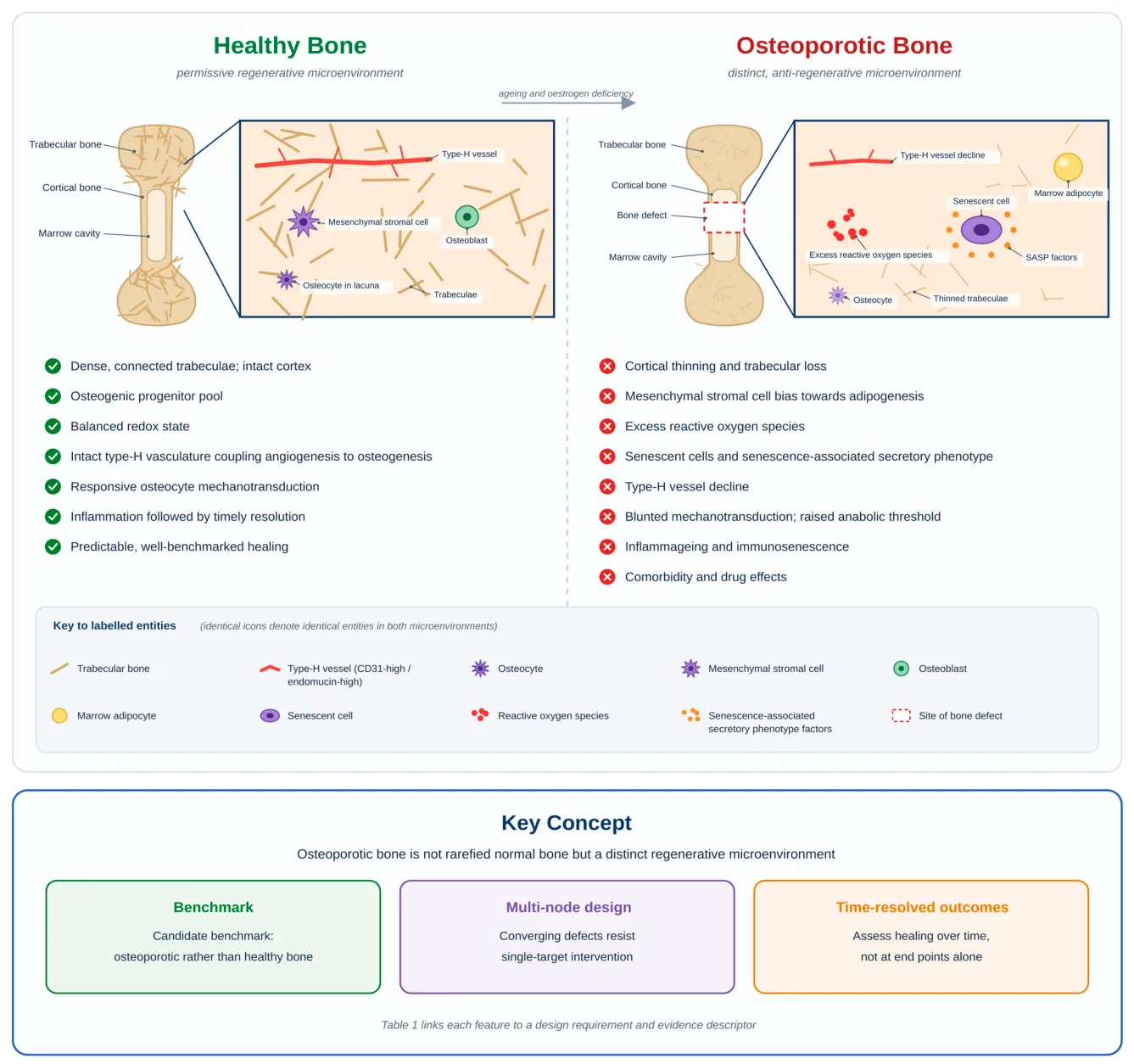
Osteoporotic bone as a distinct regenerative microenvironment rather than merely rarefied normal bone. The whole-bone diagrams identify the cortical bone, trabecular bone, and marrow cavity, and the boxed metaphyseal region is magnified in each panel. Within the magnified fields, every entity is labelled: trabeculae, type-H vessels (CD31-high/endomucin-high), osteocytes, mesenchymal stromal cells, osteoblasts, marrow adipocytes, senescent cells, reactive oxygen species, and senescence-associated secretory phenotype factors. Identical icons denote identical entities in the two microenvironments, so that the osteoporotic panel represents a change in state within the same compartment rather than a different tissue, and a shared key defines each icon once. The dashed outline in the right-hand panel marks the site of an osteoporotic bone defect. Ticks and crosses denote features permissive or hostile to repair. The schematic is conceptual; design requirements and evidence descriptors are given in Table 1.

Osteoporotic bone differs from healthy bone qualitatively, not only quantitatively, and each difference informs a design requirement. These alterations converge, rendering osteoporotic repair resistant to single-target interventions (Figure 2). Table 1 summarises these features, corresponding design requirements, and evidence descriptors.

The osteoprogenitor compartment is also functionally impaired. Ageing and oestrogen withdrawal redirect the bone marrow mesenchymal stromal cell (MSC) pool toward adipogenesis at the expense of osteoblastogenesis: marrow adiposity increases, while bone formation, proliferation, and RUNX2 expression decline, with a broader loss of regenerative competence [13]. Scaffolds relying on endogenous progenitors, therefore, draw on a diminished reservoir.

Cellular senescence drives age-related bone loss in mice. Genetic or pharmacological elimination of senescent cells or suppression of their secretory phenotype with a Janus kinase inhibitor increases bone mass and improves microarchitecture [14]. In humans and young oestrogen-deficient mice, senescence markers and senescence-associated secretory phenotype (SASP) components are elevated in bone, and oestrogen deficiency and senescence act as partially independent drivers of bone loss [15].

Senescent osteocytes, the most abundant and longest-lived bone cells, are strongly implicated; systemic clearance of senescent cells prevents bone loss at the spine and femur through effects on both osteoblasts and osteoclasts [14]. As summarised by Hofbauer et al., osteocyte-restricted senolysis is only partially effective, and transplanted senescent cells can induce host osteocyte senescence and bone loss in young mice; both observations come from that review rather than a single primary study [16]. The SASP—interleukin-6, interleukin-1, tumour-necrosis-factor-α, matrix metalloproteinases, and chemokines—links senescence to chronic inflammation, impaired osteogenesis, and disturbed angiogenesis [16]. Therefore, the environment into which a construct is implanted may be actively anti-regenerative; thus, altering it, rather than merely providing a matrix, is a legitimate scaffold function.

These processes are interlocked rather than parallel. Excess reactive oxygen species (ROS) promote osteoclastogenesis, suppress osteoblast differentiation, and reinforce stromal and osteocyte senescence [12,28]. Senescent cells are a sustained source of ROS and pro-oxidant SASP factors; thus, the two form a bidirectional, self-amplifying redox–senescence axis that is increasingly recognised as a driver of age-related tissue degeneration and a therapeutic target [12].

**Figure 3:**
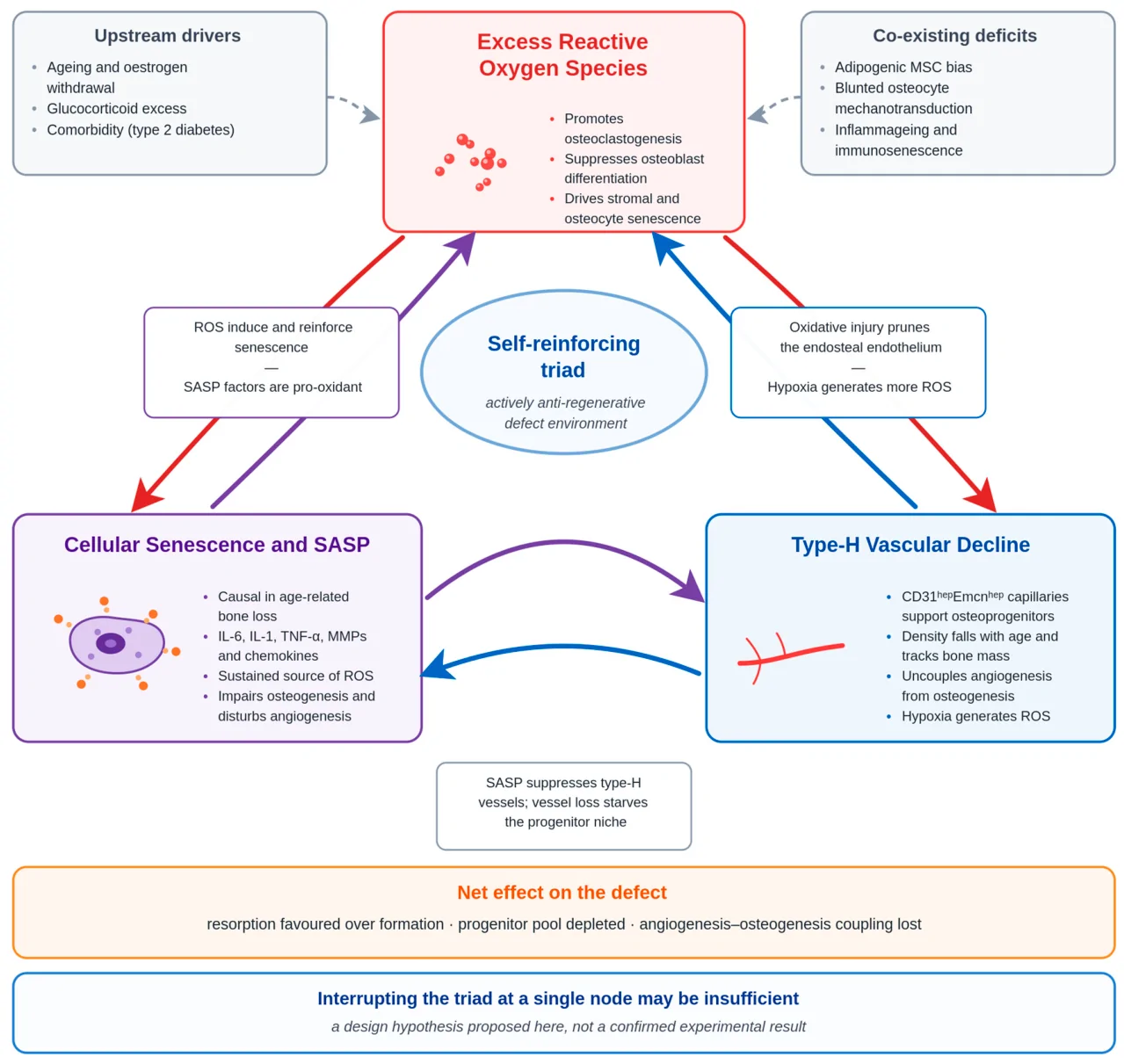
The proposed redox–senescence–type-H vascular triad. The superscript notation shown in the diagram should be read as CD31-high/endomucin-high. Reciprocal arrows indicate the mutually reinforcing relationships described in Section 3; upstream drivers and co-existing deficits are shown in grey. The triad, and the inference that interruption at a single node may be insufficient, are author-proposed syntheses rather than confirmed experimental results.

This circuit extends to the vasculature. Angiogenesis and osteogenesis are linked through type-H vessels, specialised metaphyseal and endosteal capillaries with high co-expression of CD31 and endomucin (CD31-high/endomucin-high) that support osterix-positive osteoprogenitors [18]. In human bone, type-H vessel density decreases with age and correlates with bone mass, although this relationship is associative rather than causative [19]. In an osteoporotic rodent model, an injectable short-fibre scaffold that combined ROS scavenging with sustained parathyroid hormone (1–34) release promoted type-H vessel formation and improved defect repair, linking redox and vascular elements in an osteoporotic context, although the co-delivered anabolic agent prevented the redox contribution from being isolated [20]. Because oxidative and senescent signalling suppress the endothelium that sustains type-H vessels, the redox–senescence axis and vascular decline may constitute a self-reinforcing triad, with each component potentially exacerbating the others, whereas hypoxia from vascular loss generates further ROS. Therefore, interrupting this triad at a single node may be insufficient, which is a design hypothesis rather than a confirmed result (Figure 3).

The mechanical arm is also disturbed. Bone adapts to load through osteocyte mechanotransduction, in which strain suppresses sclerostin and de-represses Wnt-driven bone formation, with mechanosensitive channels such as PIEZO1 among the proximal sensors. PIEZO1 deficiency in osteoblastic cells reduces bone mass and renders mice resistant to further loss under hindlimb unloading, identifying it as a major skeletal mechanosensor [29]. In aged bone, this response appears blunted: the same stimulus may produce less new bone, associated with altered osteocyte lacunar morphology and reduced connectivity [24]. The effective anabolic threshold therefore appears to rise with age, inferred from these data rather than measured directly [24], so a scaffold that loads osteocytes appropriately in young bone may not reach the strain required in aged bone.

Patients with osteoporosis rarely present in isolation, and both comorbidities and medications shape healing. Glucocorticoid excess, the commonest cause of secondary osteoporosis, suppresses osteoblast proliferation and differentiation, promotes osteoblast and osteocyte apoptosis, biases MSCs towards adipogenesis and prolongs osteoclast survival [25]; it impairs fracture healing by mechanisms distinct from oestrogen deficiency [4]. Type 2 diabetes impairs fracture healing via hyperglycaemia, accumulation of advanced glycation end-products, chronic inflammation, impaired vascularisation and angiogenesis, and a shift in osteoprogenitors towards adipogenic differentiation within the fracture callus [26]. This profile overlaps substantially with the ageing osteoporotic phenotype, though its interaction with scaffold integration remains poorly characterised. Anti-resorptive effects are agent- and context-dependent: bisphosphonates and denosumab differ in mechanism and reversibility, and by suppressing remodelling may impair implant incorporation in some settings while preserving peri-implant bone in others. In dental implant cohorts, which provide the largest body of relevant human data, low-dose bisphosphonate therapy has not been shown to increase early implant failure, whereas the effect of denosumab and the influence of either agent on long-term survival remain insufficiently documented [27]. Dental-implant findings may not generalise to orthopaedic scaffold integration.

On this reasoning, the osteoporotic microenvironment may require a scaffold addressing several of these nodes simultaneously rather than any one alone; the individual design requirements and their evidence descriptors are set out in Table 1.

## 4. Materials, Biofabrication and Mechanical Adaptation

Each material class presents trade-offs that are exacerbated by osteoporosis (Table 2). Calcium phosphate ceramics, chiefly hydroxyapatite and the more rapidly resorbable β-tricalcium phosphate, are osteoconductive and release calcium and phosphate, favouring mineralisation, but are brittle and weak in tension. Bioactive glasses contribute ionic bioactivity and angiogenic dissolution products but also display brittleness [30]. Among synthetic polymers, polycaprolactone (PCL) offers printability, ductility, and slow, predictable degradation but is hydrophobic with low intrinsic bioactivity. Polylactic acid and related polyesters degrade faster while releasing acidic byproducts that may exacerbate inflammation [31]. Hydrogels and natural biomaterials, such as collagen, gelatin, and methacrylated gelatin (GelMA), offer cell-friendly, tunable matrices suited for bioprinting and drug or cell delivery; however, they are mechanically weak [32], necessitating the use of composites or porous metals for load-bearing applications. Decellularised extracellular matrix adds biological complexity, and nanomaterials contribute catalytic and delivery surface area. No single material reconciles strength, bioactivity, controlled degradation, and immunocompatibility; therefore, composites and functional gradients dominate rational design. For osteoporosis, strontium-containing ceramic and cement systems combine osteopromotive and anti-resorptive effects and have direct disease-specific in vivo evidence, examined in Section 7.

**Figure 4:**
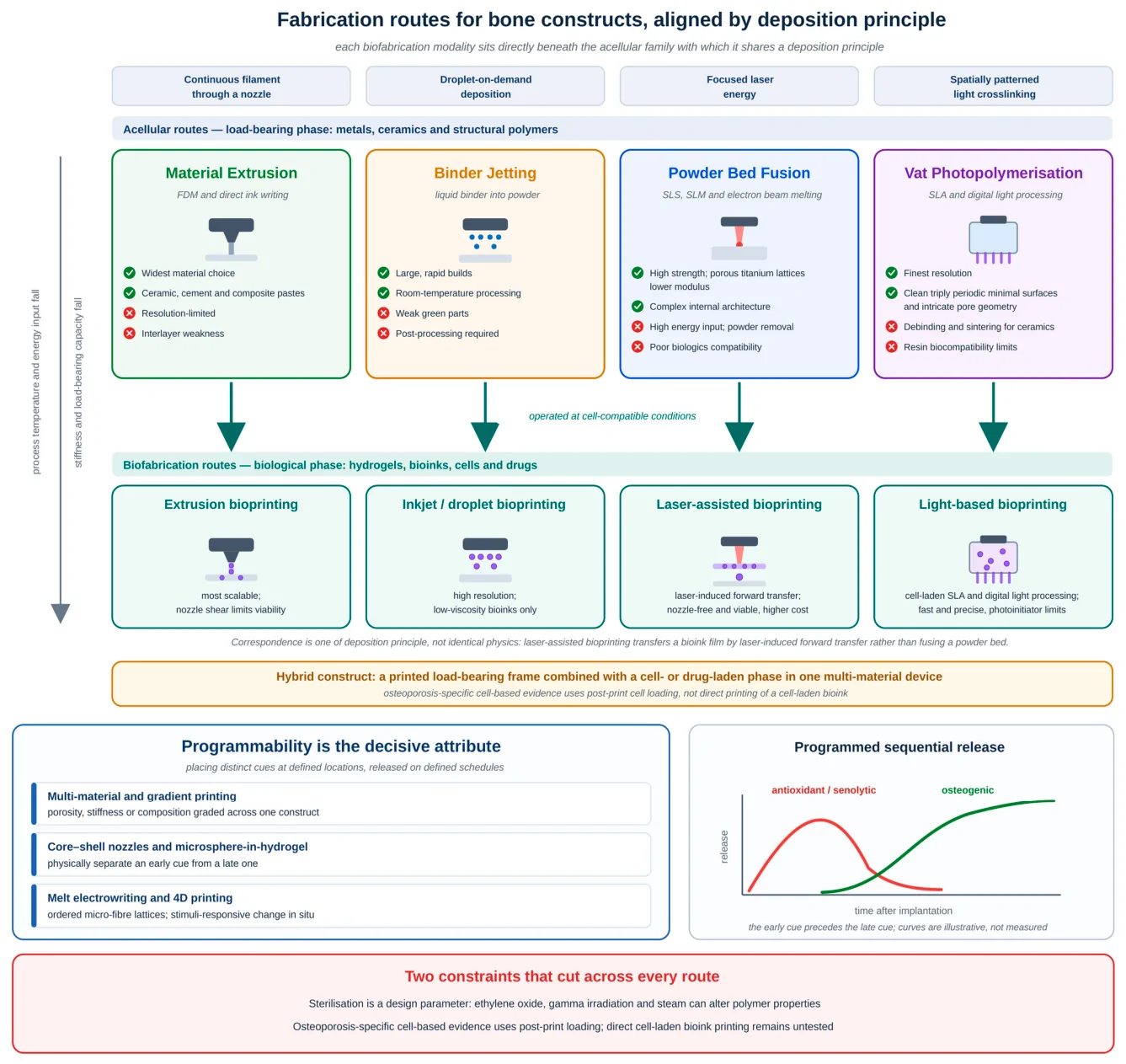
Fabrication routes for bone constructs and the programmability they confer. The upper and middle bands are aligned on deposition principle, so that each biofabrication modality sits beneath the acellular family with which it shares that principle: extrusion bioprinting beneath material extrusion, inkjet or droplet bioprinting beneath binder jetting, laser-assisted bioprinting beneath powder bed fusion, and light-based bioprinting beneath vat photopolymerisation. The correspondence is one of deposition principle rather than of identical physics, since laser-assisted bioprinting operates by laser-induced forward transfer of a bioink film rather than by fusion of a powder bed. Moving from the upper to the middle band, process temperature and energy input fall so that living cells and biologics remain viable, while stiffness and load-bearing capacity fall in parallel; the upper band therefore supplies the load-bearing phase and the middle band the biological phase, which the hybrid-construct arrow combines within a single multi-material device. Lower left: strategies achieving spatial and temporal control. Lower right: schematic release profiles for a programmed sequence; the curves are illustrative, not measured. Disease-specific cell-based evidence rests on post-print cell loading [47] rather than direct printing of a cell-laden bioink, as set out in Section 4.

The printing route governs both the achievable architecture and embeddable biology. Additive manufacturing for bone can be divided into acellular and cell-laden approaches; the acellular routes, which process the strongest and most load-relevant materials, fall into four families [40]. These are material extrusion, powder bed fusion, vat photopolymerisation, and binder jetting; their principal advantages and limitations are summarised in Figure 4. Material extrusion is the most flexible in terms of material choice but is limited by resolution [30,41]. Powder bed fusion underlies porous titanium lattices, whose reduced modulus mitigates stress shielding at the cost of limited biocompatibility [6,40]. Vat photopolymerisation achieves the finest resolution and reproduces geometries such as triply periodic minimal surfaces, although resin chemistry can restrict biocompatibility [40]. Binder jetting allows large, rapid, room-temperature builds but yields weak green parts [40]. No single route fulfils all the requirements of an osteoporotic construct.

Biofabrication, used here for approaches that place living cells and biologics within a printed construct, whether deposited in the bioink or introduced after fabrication, trades mechanical performance for biological content, particularly in cell-laden hydrogels, and its modalities differ chiefly in how the bioink is deposited [42]. These modalities are not a separate technology from the acellular families above, but the same four deposition principles—extrusion, droplet, laser, and light—operated within the temperature, shear, and energy limits that living cells tolerate; the mechanical performance forfeited in doing so is why load-bearing and biological functions are usually assigned to different phases of a single hybrid construct (Figure 4). Extrusion, inkjet/droplet, laser-assisted, and light-based modalities differ in resolution and achievable cell density.

The decisive attribute is not any single modality but the capacity to position distinct materials and cues at defined locations and to release them on defined schedules. Multi-material and gradient printing can grade porosity, stiffness, or composition across one construct, while core–shell nozzles and microsphere-in-hydrogel architectures physically separate an early cue from a late one. The antioxidant-then-osteogenic response of cerium-bearing bioactive-glass scaffolds [35] relies on fabrication-level programmability, as does the senolytic-then-osteoinductive release from an injectable, non-printed bioglass-microsphere-in-GelMA construct [37]—programmed sequential release achieved by formulation rather than by printing. Melt electrowriting adds highly ordered micro-fibre lattices that impart mechanical order and topographic guidance to otherwise soft matrices [43], while four-dimensional printing uses stimuli-responsive materials to change shape or trigger release after implantation [44]. Self-fitting constructs illustrate the surgical appeal of this approach for irregular defects. In a recent example, extrusion-printed polylactide-co-trimethylene carbonate scaffolds nanoengineered with polydopamine nanoparticles were programmed into a compact form and then deployed intraoperatively by near-infrared (NIR) irradiation at low power intensity (0.76 W cm^−2^), recovering more than 99% of their programmed shape within 30 s and conforming to the defect [45]. In critical-sized rabbit cranial defects, the composite scaffolds supported near-complete bone regeneration at 6 and 12 weeks on micro-computed tomography, in contrast to the unmodified polymer [45]. Conformal, minimally invasive fitting is attractive precisely where bone stock is poor and press-fit stability is unreliable; however, this construct was evaluated in healthy, non-osteoporotic animals at a non-load-bearing cranial site, and self-fitting scaffolds remain untested in any osteoporosis-defined model. Four-dimensional strategies therefore remain largely experimental [44]. Patient-specific digital workflows convert computed tomography data into printable models, which are increasingly optimised computationally before manufacture [46]. Hybrid strategies combining printing with freeze-drying, gas foaming, or electrospinning add features that printing alone cannot achieve.

Sterilisation compatibility also constrains material selection from the outset [39]; because it belongs with the other regulatory and manufacturing constraints, it is considered with the translational barriers in Section 8.

The mechanical design problem is twofold: the construct must restore immediate stability in poor-quality host bone while avoiding stress shielding that accelerates resorption of that same bone. Table 3 summarises the principal strategies and the supporting evidence. Solid high-modulus metallic implants do not meet the second requirement; porous and functionally graded architectures lower the effective stiffness towards bone-like values while preserving strength. Computational and biomechanical studies support this approach for porous printed titanium [6], and the clinical adoption of porous cages has been associated with reduced subsidence in a single retrospective clinical cohort [8]. This association does not establish reduced stress shielding as the mechanism.

**Figure 5:**
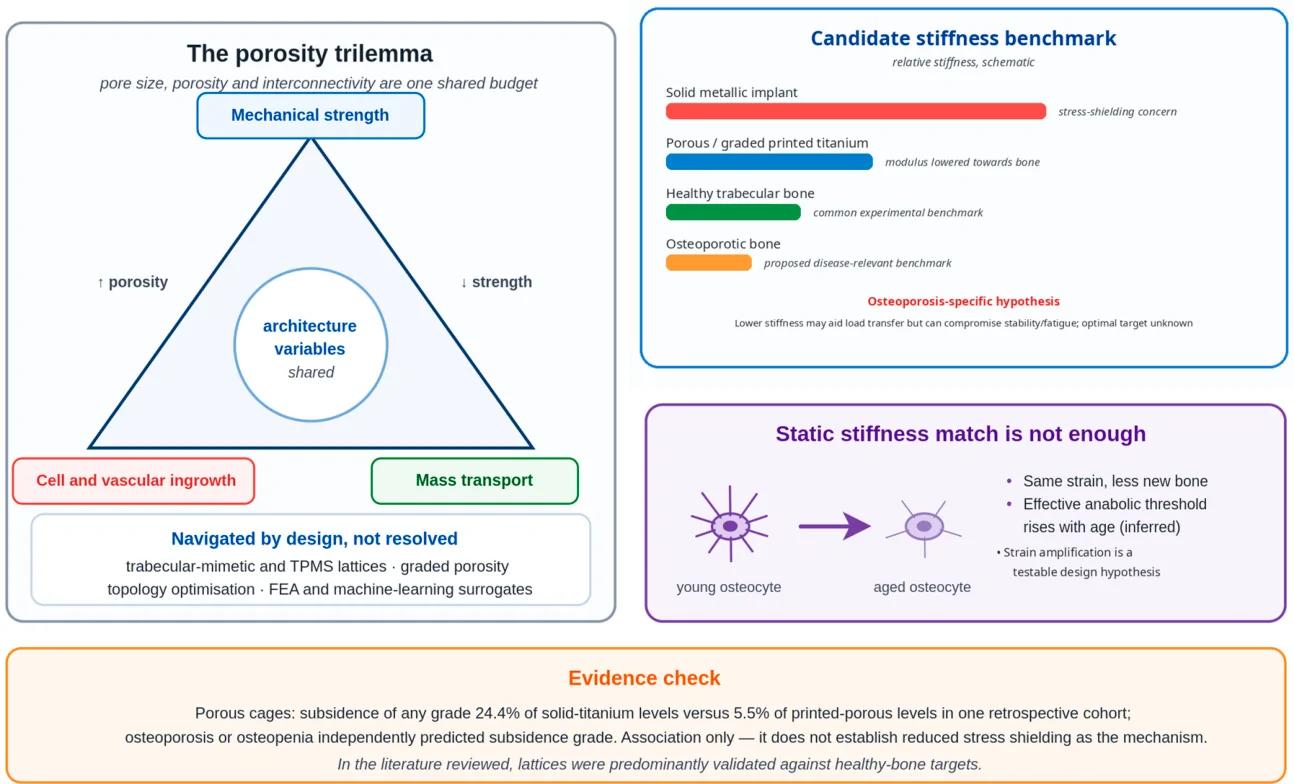
Mechanical design for osteoporotic bone. Left: the trilemma in which pore size, porosity, and interconnectivity are a shared budget across mechanical strength, cell and vascular ingrowth, and mass transport. Upper right: relative stiffness of implant and bone, schematic and not to scale; an osteoporotic-bone benchmark is proposed as disease-relevant, but the optimal stiffness remains unestablished because load-transfer benefits must be balanced against construct stability and fatigue resistance. Bars indicate rank only, not modulus values. Lower right: age-related blunting of osteocyte mechanotransduction makes strain amplification a testable hypothesis rather than an established design rule. Subsidence figures are from a single retrospective clinical cohort and establish association only.

Pore size, porosity, and interconnectivity govern a recognised trilemma between mechanical strength, cell and vascular ingrowth, and mass transport, in which increasing porosity to favour vascularisation sacrifices strength [48]. In interpreting this literature, porosity must itself be specified at two scales. Designed macroporosity is the interconnected pore network defined in the digital model and reproduced by the printing path; it dominates the behaviour of metallic and ceramic lattices, and it is the porosity to which reported pore-size optima usually refer. Intrinsic material micro- and nanoporosity arises instead from the material and the process—for example, sintering micropores in ceramics and solvent- or foaming-derived micropores in polymers—while in hydrogels the effective ‘porosity’ at the cell scale is largely the mesh of the swollen polymer network rather than the printed channel architecture. Nominally similar porosity values are therefore not directly comparable across metallic, ceramic, polymeric and hydrogel constructs. Functionally graded architectures vary designed porosity spatially—for example, radially, from a denser cortical-like shell to a more porous trabecular-like core—to reconcile load-bearing capacity with ingrowth, and hierarchical designs superimpose process-derived micro- and nanoporosity on printed macropores so that vascular ingrowth, mass transport and cell attachment are each served at their appropriate length scale [46,48]. These distinctions are particularly relevant to osteoporotic bone, in which the trabecular compartment is preferentially lost (Section 3), so that both the stiffness target and the pore architecture of the construct differ from healthy-bone benchmarks. Trabecular-mimetic and triply periodic minimal-surface lattices, graded-porosity designs, topology optimisation, and finite-element analysis navigate this trilemma. Computational optimisation increasingly uses machine-learning surrogates to predict tissue ingrowth, although outputs still require in vivo validation, and inter-laboratory reproducibility remains inconsistent [46]. However, two osteoporosis-specific hypotheses remain largely untested. Matching stiffness more closely to osteoporotic than healthy bone might reduce shielding of residual bone, but excessive compliance could compromise stability or fatigue resistance; no study reviewed here defines the optimal load-bearing trade-off. Likewise, because osteocyte mechanotransduction is blunted with age [24], deliberate strain amplification is a testable hypothesis rather than an established rule. Most reviewed lattices were benchmarked against healthy-bone targets (Figure 5).

## 5. Redox Regulation and Senescence Modulation

The mechanistic rationale for the strategies in this section—the self-amplifying redox–senescence loop and its proposed coupling to type-H vascular decline—is schematised in Figure 3. Two cautions apply throughout, and Table 4 presents the evidence category and directness for each approach. Most evidence is in vitro or small animal, with large animal and human data specific to osteoporotic defects being scarce or absent, and the processes manipulated are homeostatic as well as pathological, so mistimed intervention may harm as readily as benefit. These cautions are particularly relevant to redox regulation and senescence modulation, which operate along the same self-reinforcing axis.

Excess reactive oxygen species (ROS) promote osteoclastogenesis, impair osteoblast function, and reinforce cellular senescence [28]. Buffering the local redox environment is therefore a rational scaffold function, and cerium oxide nanoparticles (nanoceria) are among the most studied redox-active materials. Their reversible Ce^3+^/Ce^4+^ surface chemistry imparts superoxide dismutase- and catalase-mimetic activity. In 3D-printed bioactive-glass scaffolds, nanoceria produced early antioxidant and anti-inflammatory effects, followed by enhanced osteogenic activity in a rat model [35]. Digital-light-processing-printed hydroxyapatite scaffolds containing 5 wt% cerium-bearing mesoporous bioactive-glass nanoparticles also improved mechanical strength, antioxidant capacity, and osteogenesis in a rat calvarial critical-sized defect model [36]. However, nanoceria can be pro-oxidant in acidic microenvironments; dose–response, long-term biodistribution, and clearance remain poorly defined, and no study has tested these materials in human osteoporotic bone. The inverted-U is therefore a design heuristic rather than a demonstrated osteoporotic dose–response.

As senescent cells causally drive age-related bone loss in mice, locally reducing their burden or attenuating their senescence-associated secretory phenotype (SASP) is a logical approach. Biomaterials targeting senescent cells have become an identifiable field of study [53]. The preclinical pharmacological basis is well developed: dasatinib plus quercetin (D+Q) improves physical function and increases lifespan in aged mice, even when initiated late in life [49].

Human skeletal evidence is limited but informative. In a phase 2 randomised controlled trial of intermittent D+Q in 60 postmenopausal women, the primary endpoint, 20-week percentage change in C-terminal telopeptide of type 1 collagen, did not differ between groups (median −4.1% [−13.2, 2.6] for D+Q versus −7.7% [−20.1, 14.3] for control, *p* = 0.611). Procollagen type 1 N-terminal propeptide increased by 16% relative to control at two weeks (*p* = 0.020) and four weeks (*p* = 0.024), but not at 20 weeks (−9%, *p* = 0.149); the largest response in women with the highest senescent-cell burden was exploratory [17]. This transient anabolic signal with a null primary endpoint is consistent with a time-dependent, heterogeneous response, but the single non-dose-ranging regimen cannot establish a dose–response shape. Local, temporally programmed delivery therefore remains a rationale to test rather than a demonstrated superior strategy.

Local senescent-cell clearance in a post-traumatic joint-injury model reduced degeneration and created a pro-regenerative environment [50]. This directly supports local senescence modulation in that non-osteoporotic injury model, but only indirectly informs osteoporotic bone-defect repair. The most relevant proof of concept combines senolysis and osteoinduction in a defect animal model. Wang et al. demonstrated that oestrogen deficiency-induced bone loss in middle-aged rats was accompanied by SASP-driven inflammation and the accumulation of senescent MSCs. Systemic administration of dasatinib combined with quercetin ameliorated osteoporosis and restored MSC functions. For scaffold design, a sequentially releasing construct of mesoporous bioglass microspheres loaded with BMP-2 within a dasatinib- and quercetin-laden 10% GelMA hydrogel, implanted into a 2.5 mm distal femoral defect, improved osteoporotic bone regeneration by clearing senescent cells while delivering an osteogenic stimulus [37].

Several cautions are important. Senescence is not uniformly deleterious; transient senescence contributes to normal tissue repair, and untimed clearance can impair healing. Local delivery does not guarantee exclusive local action. Long-term systemic safety remains unestablished, and there is no human evidence demonstrating that scaffold-delivered senolytics improve osteoporotic defect healing. Their appraisal must distinguish systemic-drug evidence from the far thinner scaffold-delivery data [53].

## 6. Osteoimmunomodulation and Vascularisation

**Figure 6:**
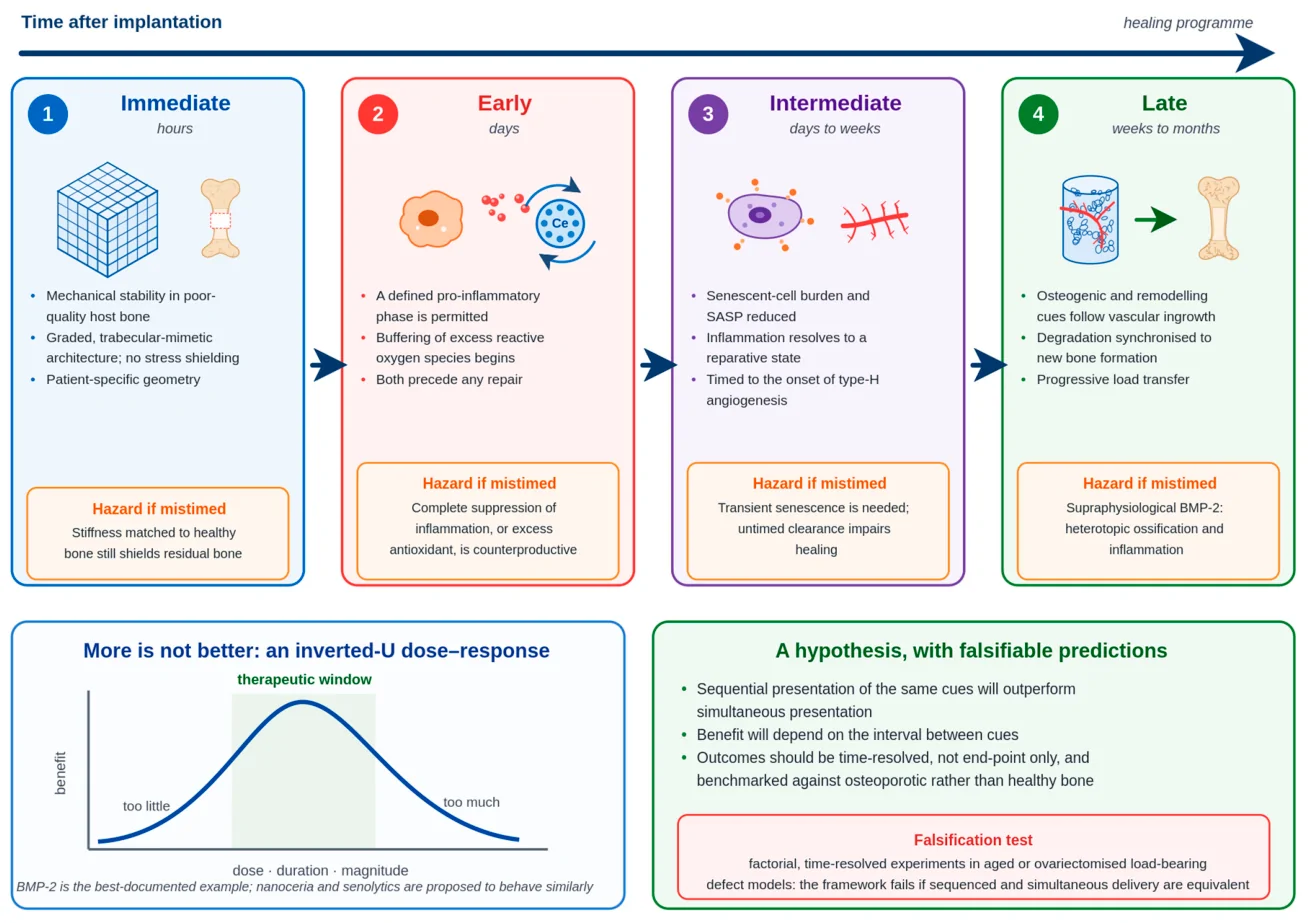
The integrative, time-sequenced design framework proposed in Section 9. Each phase carries a distinct objective and a corresponding hazard if the cue is delivered outside its window. Lower left: the inverted-U dose–response, shown schematically. Lower right: the predictions the framework generates and the experiment that would falsify it. All elements are schematic, and the sequence is untested as a whole.

Bone healing is immunologically orchestrated, and scaffold implantation is itself an immune event. Macrophage states are dynamic and context-dependent; the M1/M2 dichotomy oversimplifies their function, and repair requires an early inflammatory phase followed by timely resolution and reparative, pro-osteogenic and pro-angiogenic activity [21,54]. The temporal window within which this resolution must occur, and its position in the overall design sequence, are schematised in Figure 6 (Section 9). Printed scaffold architecture can bias macrophage behaviour and enhance osteogenesis without pharmacological intervention [10,55]. Disease-specific evidence is limited: a hierarchical 3D-printed porous Ti6Al4V scaffold releasing icariin and Mg^2+^ shifted macrophages towards M2 through Notch1 inhibition and improved peri-implant bone formation in osteoporotic rats [23]—a single small-animal osseointegration study, not defect repair or human evidence. Strontium-functionalised biomaterials also exert osteoimmunomodulatory effects [51], and zinc/strontium co-assembled hydroxyapatite has demonstrated programmed immune-modulating and osteogenic cue presentation [52].

Regardless of the specific cue, the osteoporotic starting point is abnormal because inflammageing and immunosenescence bias the system toward persistent inflammation [22]. The design objective for an osteoimmunomodulatory scaffold is therefore more than the delivery of an anti-inflammatory payload; it is to restore the temporal sequence of inflammation and resolution. Excessive or premature immune suppression is a genuine hazard because the early inflammatory phase is a prerequisite for regeneration.

A scaffold in an osteoporotic defect also meets the adaptive immune system, for which the axis formed by receptor activator of nuclear factor-κB (RANK), its ligand RANKL, and osteoprotegerin (OPG) is the principal molecular link between the two. Activated T and B lymphocytes produce RANKL and cytokines, such as tumour necrosis factor-α and interleukin-17, which promote osteoclastogenesis, whereas regulatory T cells and subsets producing interleukin-4 and interferon-γ inhibit this process [54]. Ageing alters these immune populations by reducing the naive T-cell pool and expanding memory and senescent subsets [22]. The defect is thus colonised by an immune system already biassed towards resorption; therefore, constructs modulating only the macrophage phenotype address part of a broader dysregulation.

Surface topography and roughness, pore size and geometry, substrate stiffness, and degradation kinetics are the variables that additive manufacturing controls most precisely, and printed constructs can present these cues in defined spatial and temporal patterns [21]. However, two cautions temper this conclusion. First, the pore and stiffness parameters that optimise immune resolution may not be the same as those that optimise mechanical performance or vascular ingrowth; therefore, osteoimmunomodulation competes with the mechanical trilemma for the same design variables [48]. Second, most supporting evidence is derived from healthy or young models; how an inflamed, immunosenescent host responds to identical cues is uncharacterised, and designs validated in normal bone cannot be assumed to perform similarly in osteoporotic bone.

Vascularisation frequently limits healing in larger defects and is further compromised in osteoporotic bone by decline of osteogenesis-coupled type-H vasculature [18,19] and redox/senescence signalling. Strategies include vessel-permissive pore architecture, angiogenic factors, early angiogenic cues before osteogenic cues, oxygen-generating materials, and pre-vascularised constructs. Because endothelial and osteogenic programmes are coupled, type-H-like vascular support is particularly relevant. However, interventions targeting Notch or hypoxia-inducible factor signalling have not been evaluated in osteoporotic defect models in the studies reviewed. General vascular density should not be equated with bona fide type-H endothelium: claims require appropriate CD31-high/endomucin-high phenotyping. As with BMP-2 [7], angiogenic cues require dose and timing control; prolonged stimulation may yield disorganised vasculature, while direct scaffold evidence in osteoporotic defects remains limited.

## 7. Preclinical Models and the State of Clinical Evidence

The osteoporosis-specific in vivo evidence base rests heavily on ovariectomised (OVX) rodents, with glucocorticoid-induced and senescence-accelerated models used less often; strontium-substituted ceramics and cements provide some of the clearest disease-specific in vivo evidence. Table 5 summarises representative studies, several of which utilised conventional fabrication methods rather than additive manufacturing, a limitation discussed further in Section 11. In an osteoporotic rat metaphyseal femoral bone defect model, bioceramics composed of strontium-doped hydroxyapatite whiskers, designed for slow and sustained release, promoted osteogenesis-coupled blood vessel formation within a week. At 12 weeks, these scaffolds generated more new bone within the defect and at the implant–bone interface than strontium-free scaffolds, achieving selected skeletal outcomes comparable to systemic strontium ranelate with lower measured systemic exposure [33]. This does not establish a clinical safety advantage. In a critical-sized femoral metaphyseal defect in OVX rats, strontium-modified calcium-phosphate cement combined with a single low local dose of BMP-2 (5 µg) enhanced defect healing at eight weeks more effectively than either unmodified or strontium-modified cement alone [34].

Beyond strontium, the co-delivery of BMP-2 with osteoprotegerin from 3D-printed Ti6Al4V scaffolds improved osseointegration under osteoporotic conditions, combining osteogenic and anti-resorptive signals to address both arms of remodelling imbalance [9]. However, these studies have limitations. OVX rodents reproduce oestrogen-deficiency bone loss but not the full human syndrome of ageing, comorbidity, immunosenescence, and altered biomechanics. Defects are typically small and non-load-bearing, follow-up is short, and males—who face the larger projected rise in hip fracture [2]—are rarely included. A systematic review and meta-analysis of 3D-printed scaffolds in animal femoral and tibial defects confirmed a signal of efficacy alongside substantial heterogeneity and risk of bias, which limit confident synthesis [57]. Constructs addressing several axes simultaneously have recently been reported in osteoporotic models: a 3D-printed titanium scaffold with a photothermal-responsive antioxidant hydrogel coating targeting impaired osteogenesis, inflammation, oxidative stress, and senescence [56], and a 3D-printed polycaprolactone/hydrogel scaffold combining multi-enzyme reactive-oxygen-species scavenging with macrophage regulation [38]. Both are small-animal studies, but they indicate that the multi-axis design logic proposed here is being tested experimentally. Large-animal osteoporotic models, aged and comorbid models, and load-bearing sites remain notably underrepresented, which is a major weakness in the preclinical literature.

Within the literature search described in Section 2, we did not identify direct human evidence for 3D-printed or biofabricated scaffolds in osteoporosis-defined cohorts. The clinical use of printed devices exists largely outside osteoporosis-defined cohorts, most extensively with porous titanium interbody cages, where patient-reported outcomes and revision rates did not differ appreciably between cage types [8]. In long-bone reconstruction, 3D-printed porous Ti6Al4V scaffolds have been applied prospectively to critical diaphyseal defects of the lower limb within a staged induced-membrane (Masquelet) technique after debridement for osteomyelitis or aseptic non-union (10 patients, 11 defects, mean defect length 12.2 cm, and mean follow-up 23.0 months), with progressive peri-scaffold bone formation, no scaffold breakage, and one revision for tibial varus deformity [58]. Printed bioresorbable scaffolds have been used for maxillofacial reconstruction. A scoping review identified 13 clinical reports (six case reports, five case series, one prospective study, and one randomised trial) that reported favourable bone formation and dental-implant support, but also complications such as graft exposure, wound dehiscence, and infection [59]. Neither body of evidence was generated in patients selected for osteoporosis, and neither evaluated the redox, senescence, or immunomodulatory strategies discussed previously. No completed human trial with reported results of the multifunctional constructs central to this review was identified within the searches described in Section 2.

## 8. Translational Barriers

The gap between preclinical promise and clinical use remains substantial and is not primarily biological (Table 6). Reproducibility and batch consistency are demanding for printed constructs and especially bioinks [32]. Cell-laden products add sourcing, potency, viability and shelf-life constraints. Sterilisation is an under-reported constraint across fabrication routes: in polylactic acid-based devices, ethylene oxide, gamma irradiation and steam autoclaving can each alter physical, chemical, mechanical and biocompatibility properties, and steam sterilisation is unsuitable for biomaterials sensitive to thermal or hydrolytic degradation [39]. Sterilisation should therefore be fixed early as a design parameter rather than treated as an afterthought, particularly for multi-material combination products that incorporate drugs or living cells. Acellular constructs require a terminal sterilisation method compatible with their components [39], whereas viable-cell products require pre-sterilised components and aseptic manufacture. Scalability, cost, surgical handling and integration with conventional fixation also influence adoption but are often neglected in laboratory studies.

Long-term considerations include the alignment of degradation kinetics with bone regeneration, ensuring mechanical fatigue resistance at load-bearing sites, and addressing the immunogenicity of biological components. Tumorigenicity is a recognised safety concern when using highly proliferative cells. Stem cell-derived products have been shown to produce tumours in animal models and, in isolated clinical reports, in patients. Tumorigenicity assessment is therefore an important component of preclinical safety evaluation for relevant stem-cell-derived products [60]. Regulatory classification also presents a barrier, as many constructs discussed here are combination products that span the device, medicinal product, and advanced therapy frameworks. The relevant definitions, oversight bodies, and approval pathways vary considerably across different jurisdictions. For example, the European Union regulates tissue-engineered and combined products within a single advanced therapy framework, whereas in the United States, constructs combining devices, drugs, or biological constituents may be regulated as combination products, with lead jurisdiction determined by the primary mode of action [61]. Therefore, classification depends on product composition and function rather than on a single universal pathway. Point-of-care printing sharpens this: hospital-based manufacture can bring manufacturer-like quality and process responsibilities into the clinical setting, depending on the jurisdiction and applicable pathway [62]. Both the technology and regulatory oversight for bioresorbable point-of-care implants are still evolving [62]. Collectively, these constraints are as influential in determining whether multifunctional constructs reach patients as any aspect of biological design.

## 9. An Integrative, Time-Sequenced Design Framework

A central hypothesis emerges: an osteoporotic scaffold may depend less on the number of biological functions it carries than on their coordination in time, dose, and space. The four axes are framed as potentially sequential, partly overlapping elements of one healing programme, not as an established design prescription.

In the hypothesised sequence, the construct would first provide mechanical stability while limiting stress shielding; graded architectures could be benchmarked against osteoporotic bone, subject to stability and fatigue constraints. Early redox buffering should accompany, rather than abolish, the necessary inflammatory phase. As inflammation subsides, time-limited senescence and immune modulation could be paired with vascular support, on the hypothesis that senescent and inflammatory signalling contributes to impaired osteogenesis-coupled vascular function. Osteogenic and remodelling cues would follow vascular ingrowth and progenitor recruitment, with degradation synchronised to new bone formation for progressive load transfer. Each phase and the hazard of mistiming it are summarised in Figure 6.

Individual elements are experimentally feasible, but the complete sequence remains a proposed model. It generates testable predictions: that the sequential presentation of the same cues will outperform simultaneous presentation, and that the benefit will depend on the interval between them. Factorial, time-resolved experiments in aged or ovariectomised load-bearing defect models would falsify the framework if sequenced and simultaneous delivery proved equivalent. Several published constructs have already separated functions temporally within one scaffold: the sequential antioxidant and osteogenic activities of cerium-oxide/bioactive-glass systems [35,36], co-delivery of local senolysis with delayed osteoinduction using a sequentially releasing bioglass–GelMA construct in an ovariectomised defect [37], and programmed, stepwise presentation of immune-modulating and osteogenic cues from dual-ion hydroxyapatite [52].

Increased biological activity is not intrinsically better; many interventions may follow an inverted-U, with efficacy confined to a window of dose and time and counterproductive outside it. BMP-2 is a well-documented example of this phenomenon [7]. The senolytic trial discussed in Section 5 is consistent with the timing- and selection-dependence of this model, although it tested a single regimen and does not itself demonstrate a dose–response shape [17]. Therefore, the critical design variables are likely to be temporal, quantitative, and spatial, rather than the number of bioactivities. Two corollaries follow: outcomes should be time-resolved rather than end-point only, and benchmarks osteoporotic rather than healthy, since constructs performing well in young bone may not meet the raised anabolic threshold of an osteoporotic defect.

## 10. Future Research Priorities

Progress requires specific and testable work. A defensible minimum validation package for a multifunctional construct begins with the model: an osteoporosis-defined animal with a healthy comparator, a clinically relevant load-bearing defect, and justified sex selection or the inclusion of both sexes. To this, it should add time-resolved biological endpoints together with mechanical and fatigue testing. Follow-up should be long enough to assess degradation and integration before large-animal translation. Key priorities include standardised, validated osteoporotic scaffold-testing models, particularly those incorporating aged and comorbid (diabetic, glucocorticoid-exposed) large animal models and load-bearing defects, to reduce reliance on young adult ovariectomised (OVX) rodents. Sex-specific analyses are essential, as most models and mechanisms have been studied in females, despite the steepest projected increase in fracture incidence occurring in men [2]. Patient-specific mechanical and biological profiling should be used to tailor stiffness targets and payloads to individual bone quality and comorbidities. Long-term degradation and fatigue studies at clinically relevant load-bearing sites are needed, as are head-to-head comparisons using standardised, time-resolved outcomes and agreed reporting standards, given the fragmented literature [57]. Further priorities are cost-effectiveness analyses, early phase trials with explicit osteoporotic selection, and multimodal, temporally programmed scaffolds whose release kinetics match the healing phases described above, with biomarkers of redox status, senescent-cell burden, and type-H ingrowth guiding selection and monitoring. The reporting of negative, null, and dose–response results is particularly important, as is the routine inclusion of healthy bone comparators.

## 11. Limitations

This is a narrative rather than a systematic review: no protocol was registered, study selection was not duplicated, no formal risk-of-bias assessment was conducted, and no meta-analysis was performed; therefore, selection and interpretation bias cannot be excluded. Only publications in English were included in this review. Earlier foundational work was incorporated through citation tracking, rather than systematic retrieval. No PRISMA-style deduplication or screening log was maintained, so negative search findings mean ‘not identified within the searches described,’ not exhaustive proof of absence. The available evidence base imposes further constraints: osteoporosis-specific in vivo research is dominated by young adult ovariectomised rodents with small, non-load-bearing defects and short follow-up periods. Male subjects are largely absent, and much of the supporting redox and immunomodulatory evidence is derived from non-osteoporotic animal models. Disease-specific cell-based biofabrication evidence relies on post-print cell loading rather than direct printing of cell-laden bioink [47]. In the searches described in Section 2, we identified no construct in which a cell-laden bioink was printed directly and then evaluated using an osteoporosis-defined defect model. Several constructs providing osteoporosis-specific evidence, such as strontium-doped bioceramics and cements and senolytic-releasing hydrogels, were produced by conventional fabrication rather than additive manufacturing; therefore, their findings inform scaffold design for osteoporosis more directly than they validate additive manufacturing. Some comorbidity and anti-resorptive evidence discussed in Section 3 is derived from the fracture-healing and dental-implant literature rather than scaffold studies, making its extrapolation to printed constructs only inferential. Figure 1, Figure 2, Figure 3, Figure 4, Figure 5 and Figure 6 are schematic and conceptual: the release profiles, stiffness comparisons, and dose–response curves shown are illustrative and are not derived from measured experimental or clinical data. Finally, the time-sequenced framework of Section 9, the redox–senescence–vascular triad on which it rests, and the inverted-U argument are author-proposed syntheses; individual elements are supported by the cited literature, but the framework as a whole is untested and should be read as a hypothesis generating testable predictions rather than an established design standard.

## 12. Conclusions

The principal observations of this review are as follows:

▯ Osteoporotic bone defects represent a combined mechanical and biological challenge that scaffolds designed and validated for healthy bone may not fully address.
▯ The most robust evidence is mechanical and materials-based: porous printed constructs reduce effective stiffness in computational and biomechanical analyses [6] and have been linked to reduced subsidence in a single retrospective clinical cohort study [8].
▯ Strontium-functionalised ceramics and cements, which combine osteogenic and anti-resorptive activities, enhance osteogenesis in osteoporotic rodent models [33,34]; in the study with a systemic comparator, local strontium achieved comparable selected skeletal outcomes with lower measured systemic exposure [33], but not a demonstrated clinical safety advantage.
▯ Separately, the co-delivery of BMP-2 with OPG from printed Ti6Al4V improved osseointegration under osteoporotic conditions [9].
▯ Redox, senescence, and osteoimmunomodulatory strategies remain in the in vitro and small-animal research stages, with few exceptions; a phase 2 randomised trial of systemic senolytic therapy in postmenopausal women did not achieve its primary bone-resorption endpoint, although its transient anabolic signal and exploratory effect modification by senescent cell burden are informative for scaffold design [17].
▯ Within the searches described in Section 2, no completed clinical evaluation with reported results of a multifunctional scaffold in an osteoporosis-defined cohort was identified.

We propose—not prescribe—that coordination in time, dose, and space may matter more than maximising the number of bioactivities: a time-sequenced design interrupting the proposed redox–senescence–vascular triad at more than one node, delivering each cue in its proper window and dose. Currently, these constructs remain scientifically promising but clinically unproven for osteoporotic bone.

## Figures and Tables

**Table 1:** Pathological features of osteoporotic bone and the corresponding scaffold-design requirements.

Pathological Feature	Consequence for Repair	Design Requirement	Evidence Supporting the Design Requirement (Setting and Directness)
Cortical thinning, trabecular loss	Poor host bone for fixation; impaired load transfer	Patient-specific geometry; graded trabecular-mimetic architecture	Demonstrated for components (clinical/biomechanical); not for the combined requirement in osteoporosis
MSC adipogenic bias [13]	Functionally impaired osteoprogenitor pool	Reduced reliance on host progenitors; osteogenic cues	Mechanistically inferred
Senescent cells and SASP [14,15,16]	Anti-regenerative, pro-inflammatory local milieu	Time-limited local senolytic/senomorphic function	Small-animal for local delivery; human evidence is systemic-drug only [17]
Redox–senescence axis [12]	Self-amplifying ROS–senescence loop	Redox buffering + senescence modulation, combined and dosed	Author-proposed hypothesis, not directly tested †
Type-H vascular decline [18,19]	Impaired angiogenesis–osteogenesis coupling	Architecture and cues favouring type-H-like vessels	Small-animal, osteoporotic; composite intervention, redox contribution not isolated [20]
Inflammageing/immunosenescence [10,21,22]	Persistent inflammation; impaired resolution	Osteoimmunomodulation restoring inflammation–resolution timing	Small-animal, osteoporotic (single study) [23]; otherwise mechanistically inferred
Blunted mechanotransduction [24]	Reduced anabolic response to load	Stiffness benchmarked to osteoporotic rather than healthy bone; strain amplification	Author-proposed hypothesis, not directly tested †
Comorbidity and drug effects [4,25,26,27]	Further impaired osteogenesis/angiogenesis; altered remodelling	Designs robust to comorbidity; interaction studies required	Comorbidity documented clinically; scaffold interaction untested

Abbreviations: MSC, mesenchymal stromal cell; ROS, reactive oxygen species; SASP, senescence-associated secretory phenotype. † Author-proposed hypothesis: a design requirement advanced by the present authors on mechanistic grounds; no study identified by the searches described in Section 2 tests it directly, and it is offered as a testable proposition rather than as a finding. The same symbol carries the same meaning in Table 3. Evidence descriptors report study setting and directness rather than a single ranked hierarchy, and are used as follows: clinical observational, reported in human cohorts without randomised allocation; computational or biomechanical, modelling or mechanical testing without in vivo confirmation; small-animal, rodent studies specified as osteoporotic or non-osteoporotic; in vitro, cell or tissue culture only; mechanistically inferred, the requirement follows from established biology but has not itself been tested as a design requirement; and author-proposed hypothesis, as defined above. Each descriptor refers to the design requirement in the adjacent column, not to the pathological feature.

**Table 2:** Major scaffold materials: advantages, limitations, and relevance to osteoporotic bone.

Material	Advantages	Limitations	Osteoporosis Relevance	Evidence for OP Relevance
Hydroxyapatite/β-TCP	Osteoconductive; β-TCP resorbable; ion release	Brittle; weak in tension	Vehicle for Sr and osteopromotive ions [33,34]	Small-animal (OVX)
Bioactive glass: silicate bioactive glass printed as scaffolds [30,35]; mesoporous bioactive glass nanoparticles, including cerium-containing nanoparticles (Ce-MBGNs) [36]; bioactive glass microspheres as drug depots [37]	Ionic bioactivity; angiogenic dissolution; mesoporous nanoparticle forms combine reinforcement with ion and drug loading	Brittle; degradation control	Cerium oxide-reinforced printed bioactive glass gives sequential antioxidant-then-osteogenic action [35]; 5 wt% Ce-MBGNs reinforce printed hydroxyapatite [36]; bioactive glass microspheres in GelMA deliver D+Q [37]	Small-animal (non-OP) [35,36]; small-animal (OVX) for microsphere-based senolytic delivery [37]
Polycaprolactone	Printable; ductile; slow degradation	Hydrophobic; low bioactivity	Durable framework in multifunctional composites [38]	Small-animal (osteoporotic), as composite component [38]
PLA/polyesters	Printable; tunable degradation	Acidic by-products; sterilisation-sensitive [39]	Acidity may aggravate inflamed niche	Mechanistically inferred
Hydrogels/GelMA, collagen	Cell-friendly; bioprintable; tunable	Mechanically weak	Delivery of senolytics and osteoinductive factors [37]	Small-animal (OVX)
Porous metals (Ti6Al4V)	Strength; osseointegration; printable	Modulus mismatch if solid → stress shielding	Porous architecture (clinical cages [8]); dual-factor delivery, preclinical [9]	Retrospective clinical (cages) [8]; small-animal, osteoporotic [9]
dECM	Biological complexity; retained matrix cues	Batch-to-batch variability; negligible mechanical contribution	Bioactive phase within composites	Mechanistically inferred; non-OP
Redox-active nanomaterials: CeO_2_ nanoparticles (nanoceria), as free nanoparticles [35] or as Ce-MBGN [36]	Reversible Ce^3+^/Ce^4+^ surface chemistry giving SOD- and CAT-mimetic activity; high catalytic surface area; also reinforcing	Pro-oxidant in acidic microenvironments; dose–response, biodistribution, and clearance poorly defined	Local redox buffering timed to precede the osteogenic phase [35,36]	Small-animal (non-OP, rat) [35,36]; untested in human osteoporotic bone

Abbreviations: β-TCP, β-tricalcium phosphate; CAT, catalase; Ce-MBGNs, cerium-containing mesoporous bioactive glass nanoparticles; CeO_2_, cerium oxide; D+Q, dasatinib plus quercetin; dECM, decellularised extracellular matrix; GelMA, methacrylated gelatin; OP, osteoporosis; OVX, ovariectomised; PLA, polylactic acid; SOD, superoxide dismutase; Sr, strontium; Ti6Al4V, titanium–aluminium–vanadium alloy.

**Table 3:** Mechanical design strategies and supporting evidence.

Strategy	Rationale/Effect	Evidence
Porous vs. solid printed titanium	Lower effective modulus; reduced subsidence vs. solid titanium (24.4% [22/90] vs. 5.5% [4/73] levels) [8]	Retrospective clinical [8]; computational/biomechanical [6]
Functionally graded/TPMS lattices; topology + ML optimisation	Balance strength, ingrowth, mass transport	Computational/preclinical [46,48]
Stiffness benchmarked to osteoporotic bone	Potentially reduce shielding of residual bone; stability/fatigue trade-off unresolved	Author-proposed hypothesis, not directly tested †
Strain-amplifying designs	Address blunted mechanotransduction; effect untested	Author-proposed hypothesis, not directly tested †; rationale from [24]

Abbreviations: ML, machine learning; TPMS, triply periodic minimal surface. † Author-proposed hypothesis, as defined in the footnote to Table 1. Subsidence denominators are treated as spinal levels (163 levels in 124 patients), not patients.

**Table 4:** Redox-regulating, senescence-modulating and osteoimmunomodulatory approaches: evidence category/directness and key references.

Approach	Mechanism	Evidence Category/Directness	Key Refs.
Cerium oxide nanoparticles (nanoceria, CeO_2_) in printed BG or HA scaffolds, as free nanoparticles [35] or as Ce-MBGNs [36]	SOD/CAT-mimetic; sequential antioxidant→osteogenic	Small-animal (rat)	[35,36]
Local senolytic (D+Q) + BMP-2, sequential	Clear senescent MSCs; co-deliver osteoinduction	Small-animal (OVX)	[37]
Systemic senolytics and other senescence-targeting approaches	Reduce senescent-cell burden/SASP	Small-animal (rodent); one phase 2 RCT in postmenopausal women, primary bone-resorption endpoint not met. Systemic-drug evidence only; does not validate scaffold-delivered senolytics	[14,17,49]
Local senescent-cell clearance in non-osteoporotic injury	Reduce local senescent-cell burden	Small-animal, post-traumatic joint injury; not an osteoporotic defect model	[50]
Sr/dual-ion functionalisation	Osteoimmunomodulation; anti-resorptive + anabolic	In vitro/small-animal	[33,34,51,52]
Icariin/Mg^2+^ release from printed Ti6Al4V	M2 polarisation via Notch1 inhibition	Small-animal, osteoporotic (osseointegration, not defect repair)	[23]
BMP-2 + OPG co-delivery (printed Ti6Al4V)	Osteogenic plus anti-resorptive cue in one construct	Small-animal	[9]

Abbreviations: BG, bioactive glass; BMP-2, bone morphogenetic protein-2; CAT, catalase; D+Q, dasatinib plus quercetin; HA, hydroxyapatite; MSC, mesenchymal stromal cell; OPG, osteoprotegerin; OVX, ovariectomised; RCT, randomised controlled trial; Ti6Al4V, titanium–aluminium–vanadium alloy; SASP, senescence-associated secretory phenotype; SOD, superoxide dismutase; Sr, strontium.

**Table 5:** Representative in vivo studies relevant to osteoporotic scaffold design (all small-animal/rodent; osteoporosis-model status noted per row).

Model	Construct	Main Finding	Refs.
Osteoporotic rat, metaphyseal femoral defect	Sr-doped hydroxyapatite-whisker bioceramic (SrWCP)	Osteogenesis-coupled vessels at 1 wk; more bone at 12 wk; comparable to systemic Sr for measured endpoints	[33]
OVX rat, critical-sized femoral metaphyseal defect	Sr-modified CPC + single 5 µg local BMP-2	Improved healing at 8 wk; superior to CPC or Sr-CPC alone	[34]
Osteoporotic rat, osseointegration	3D-printed Ti6Al4V + BMP-2 + OPG	Improved osseointegration under osteoporotic conditions	[9]
Osteoporotic rat, intramedullary femoral implantation	Hierarchical 3D-printed porous Ti6Al4V with icariin@Mg-MOF-74	M2 polarisation; improved peri-implant bone formation and osseointegration	[23]
Rat defect; rat calvaria (non-osteoporotic)	CeO_2_-reinforced 3D-printed BG [35] and Ce-MBGN-reinforced 3D-printed HA [36]	Sequential antioxidant→osteogenic; reinforced mechanics	[35,36]
OVX rat, 2.5 mm distal femoral defect	BG-BMP2 microspheres in D+Q/GelMA (injectable, sequential)	Local senolysis + osteoinduction improved regeneration	[37]
Osteoporotic rat, femoral defect	3D-printed Ti + photothermal-responsive antioxidant hydrogel coating	Targeted impaired osteogenesis, inflammation, oxidative stress, and senescence together	[56]
Osteoporotic rat, bone defect	3D-printed PCL/gelatin–hyaluronic acid hydrogel with MgMn-LDH	Multi-enzyme ROS scavenging with macrophage regulation; improved defect repair	[38]

Abbreviations: BG, bioactive glass; BMP-2, bone morphogenetic protein-2; Ce-MBGNs, cerium-containing mesoporous bioactive glass nanoparticles; CeO_2_, cerium oxide; CPC, calcium phosphate cement; D+Q, dasatinib plus quercetin; GelMA, methacrylated gelatin; HA, hydroxyapatite; MgMn-LDH, magnesium–manganese layered double hydroxide; OPG, osteoprotegerin; OVX, ovariectomised; PCL, polycaprolactone; ROS, reactive oxygen species; Sr, strontium; SrWCP, strontium-doped whiskered calcium phosphate; Ti6Al4V, titanium–aluminium–vanadium alloy.

**Table 6:** Principal translational barriers and the research needed to address them.

Barrier	Proposed Solution/Research Need
Bioink and manufacturing variability [32]	Standardised formulations; in-process quality assurance; defined potency assays
Sterilisation and aseptic manufacture [39]	Terminal sterilisation for acellular constructs; pre-sterilised components and aseptic processing for cell-containing products
Cell sourcing, storage and shelf-life	Defined sourcing and potency criteria; point-of-care logistics
Tumorigenicity of cell-based components [60]	Formal tumorigenicity assessment within preclinical safety evaluation
Long-term degradation and mechanical fatigue	Load-bearing large-animal studies with extended follow-up
Regulatory classification of combination and point-of-care products [61,62]	Design to device, medicinal-product and ATMP frameworks from the outset; clarification of point-of-care oversight
Absent osteoporosis-specific clinical evidence	Early-phase trials with explicit osteoporotic selection and standardised outcomes
Cost, reimbursement and adoption	Cost-effectiveness analysis; integration with conventional fixation; surgical handling studies

Abbreviation: ATMP, advanced therapy medicinal product. Bracketed citations identify barriers for which published evidence is available; the proposed solutions are author recommendations rather than sourced prescriptions.

## Data Availability

No new data were created or analysed in this study. Data sharing is not applicable to this article.

## Abbreviations

ATMP, advanced therapy medicinal product; BG, bioactive glass; BMP-2, bone morphogenetic protein-2; CaP, calcium phosphate; CAT, catalase; Ce-MBGN, cerium-containing mesoporous bioactive glass nanoparticle; CPC, calcium phosphate cement; CTx, C-terminal telopeptide of type 1 collagen; dECM, decellularised extracellular matrix; D+Q, dasatinib plus quercetin; GelMA, methacrylated gelatin; HA, hydroxyapatite; MSC, mesenchymal stromal cell; NIR, near-infrared; OPG, osteoprotegerin; OVX, ovariectomised; P1NP, procollagen type 1 N-terminal propeptide; PLA, polylactic acid; RANK, receptor activator of nuclear factor-κB; RANKL, receptor activator of nuclear factor-κB ligand; ROS, reactive oxygen species; SASP, senescence-associated secretory phenotype; SOD, superoxide dismutase; Sr, strontium; Ti6Al4V, titanium–aluminium–vanadium alloy; TPMS, triply periodic minimal surface; β-TCP, β-tricalcium phosphate.
